# Evaluation of Transfer Learning with Deep Convolutional Neural Networks for Screening Osteoporosis in Dental Panoramic Radiographs

**DOI:** 10.3390/jcm9020392

**Published:** 2020-02-01

**Authors:** Ki-Sun Lee, Seok-Ki Jung, Jae-Jun Ryu, Sang-Wan Shin, Jinwook Choi

**Affiliations:** 1 Department of Biomedical Engineering, College of Medicine, Seoul National University, Seoul 03080, Korea; 2 Department of Clinical Dentistry, College of Medicine, Korea University, Seoul 02841, Korea; 3 Department of Prosthodontics, Korea University An-san Hospital, Gyung-gi do 15355, Korea; 4 Department of Orthodontics, Korea University Ansan Hospital, Gyung-gi do 15355, Korea; jgosggg@korea.ac.kr; 5 Department of Prosthodontics, Korea University Anam Hospital, Seoul 02841, Korea; koprosth@unitel.co.kr; 6 Department of Advanced Prosthodontics, Graduate School of Clinical Dentistry, Korea University, Seoul 02841, Korea; swshin@korea.ac.kr; 7 Institute of Clinical Dental Research, Korea University, Seoul 02841, Korea; 8 Institute of Medical & Biological Engineering, Medical Research Center, Seoul 03080, Korea

**Keywords:** osteoporosis screening, artificial intelligence, convolutional neural networks, dental panoramic radiographs

## Abstract

Dental panoramic radiographs (DPRs) provide information required to potentially evaluate bone density changes through a textural and morphological feature analysis on a mandible. This study aims to evaluate the discriminating performance of deep convolutional neural networks (CNNs), employed with various transfer learning strategies, on the classification of specific features of osteoporosis in DPRs. For objective labeling, we collected a dataset containing 680 images from different patients who underwent both skeletal bone mineral density and digital panoramic radiographic examinations at the Korea University Ansan Hospital between 2009 and 2018. Four study groups were used to evaluate the impact of various transfer learning strategies on deep CNN models as follows: a basic CNN model with three convolutional layers (CNN3), visual geometry group deep CNN model (VGG-16), transfer learning model from VGG-16 (VGG-16_TF), and fine-tuning with the transfer learning model (VGG-16_TF_FT). The best performing model achieved an overall area under the receiver operating characteristic of 0.858. In this study, transfer learning and fine-tuning improved the performance of a deep CNN for screening osteoporosis in DPR images. In addition, using the gradient-weighted class activation mapping technique, a visual interpretation of the best performing deep CNN model indicated that the model relied on image features in the lower left and right border of the mandibular. This result suggests that deep learning-based assessment of DPR images could be useful and reliable in the automated screening of osteoporosis patients.

## 1. Introduction

Osteoporosis is a systemic disease characterized by low bone mineral density (BMD) and micro-architectural deterioration of bone structure, thereby leading to compromised bone strength and, consequently, an increased risk of fracture [1]. Hip, spine, and wrist fractures caused by osteoporosis often lead to disorders that reduce the quality of life of the patient and, in severe cases, increase the risk of mortality [2,3]. With fast population aging and an increase in life expectancy, osteoporosis is increasingly becoming a global public health issue; it has been estimated that more than 200 million people are suffering from osteoporosis [4]. According to recent statistics from the International Osteoporosis Foundation, approximately one in three women over the age of 50 will experience osteoporotic fractures, as will one in five men over the age of 50 [4,5,6,7]. Moreover, it is expected that more people will be affected by osteoporosis in the future and, consequently, the rate of osteoporotic fractures will increase [8]. This is because the disease initially develops without any symptoms, remains undiagnosed due to scarce symptomatology, and its first manifestation is often a low-energy fracture of long bones or vertebrae [9].

Generally, osteoporosis is diagnosed by evaluating bone mineral density (BMD) measurements (expressed as a T-score) using dual-energy X-ray absorptiometry (DXA), which is considered as the reference-standard examination for BMD assessment [10,11]. However, this technique is complex, expensive, and the availability is limited for overall population diagnosis [12]. Recently, digital images of dental panoramic radiographs (DPRs) have been evaluated as cost-effective and important image data for osteoporosis screening. This is because the widespread use of panoramic radiation in dental care for elderly patients with increased life expectancy and a number of studies have demonstrated the feasibility of BMD estimation and osteoporosis screening using panoramic radiography [13,14,15,16,17,18,19,20,21,22,23].

However, previous approaches primarily relied on manually categorized feature indexes [13,14,15,16,17,18,19,20,21,22,23], such as the Gonion index, mandibular cortical index, mental index, and panoramic mandibular index, and traditional classifier called machine learning (ML) algorithms, such as support vector machine (SVM) [22] and fuzzy classifiers [23], for screening osteoporosis. Although the previously handcrafted feature indexes provided sufficient evidence for assisting osteoporosis screening using panoramic radiographs, these methods for discriminating features are of a low order and do not fully characterize the heterogeneous pattern in radiographic images. In addition, most previous studies require tedious and manual operations, such as extensive preprocessing, image normalization, and region of interest (ROI) segmentation, which can significantly affect the repeatability of the classification method. 

In the last few years, deep learning algorithms, particularly deep convolutional neural networks (CNNs) architecture, have been widely recognized as a reliable approach to learn the classification of the characteristics of features directly from original medical images [24,25]. As opposed to ML approaches that rely on explicitly classified features, deep CNNs are a class of deep neural networks that can learn high dimensional features to maximize the networks ability to discriminate abnormalities among images [26]. There are many different CNN architectures that have been designed to perform image classifications and recognitions. Each of these architectures differ in specific aspects, including the number and size of layers, the connections between these layers, and the overall network depth. Because different network architectures are best suited for different problems, and it is difficult to know in advance which architecture is the right choice for a given task, empirical examination is often recognized as the best way to make these decisions [27]. 

Although deep CNNs have been recognized as efficient tools for image classification, they require a large amount of training data, which can be difficult to apply to medical radiographic image data. When the target dataset is significantly smaller than the base dataset, transfer learning is considered a powerful technique for training deep CNNs without overfitting [28,29]. The general process of transfer learning is performed through the use of pretrained models in a two-step method as follows: First, copying the first n layers of pretrained base network on a general large dataset to the first n layers of a target network and secondly, the remaining layers of the target network are then randomly initialized and trained on a small local dataset toward the target task [28]. On the basis of the transfer learning techniques, several state-of-the-art results showed outperformance in both general image classification [30,31,32] and medical image classification [33,34,35,36]. However, a few studies have been done to develop and evaluate transfer learning-based deep CNN models for predicting osteoporosis in DPRs.

The aim of this study is to develop and evaluate the deep learning approaches for screening osteoporosis with DPR images. Using the classified panoramic radiograph images based on the BMD value (T-score), this study evaluated several different CNN models based on osteoporosis discriminating accuracy. In addition, we quantitatively evaluated the effect of transfer learning and fine-tuning of a deep CNN model on classifying performance.

## 2. Patients and Methods

### 2.1. Patients

The study was done on a total of 680 panoramic radiograph images from 680 different patients who visited the Korea University Ansan Hospital. The patients simultaneously underwent skeletal BMD examinations and digital panoramic radiography evaluations within four months, between 2009 and 2018. The subjects were classified into a non-osteoporosis group (T-score ≥ −2.5) or osteoporosis group (T-score < −2.5), according to the World Health Organization criteria [37], into which 380 and 300 subjects were assigned, respectively. The dataset was divided into training and test sets as follows: The radiographs were selected randomly, and 136 radiographs (20% of the total), 68 each from the osteoporosis and non-osteoporosis groups, were set aside as a test set. This ensured that the testing data set only contained images of novel radiographs that had not been encountered by the model during training. The remaining 544 radiographs were used for the training and validation set. This study protocol was approved by the institutional review board of the Korea University Ansan Hospital (no. 2019AS0126).

### 2.2. Data Preprocessing

**Figure 1:**
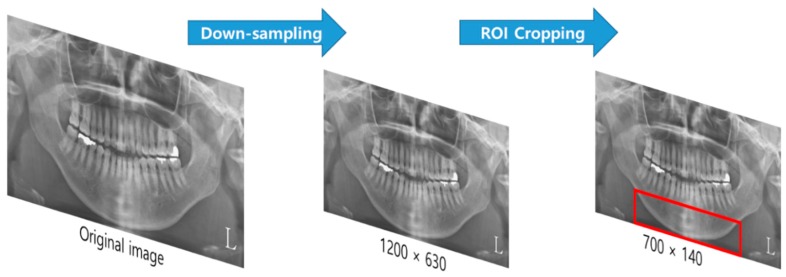
Image preprocessing for this study. The original DPRs were downsampled, and the ROI is restricted to the mandibular region below the teeth (region inside the bounding box). DPR, dental panoramic radiograph; ROI, region of interest.

The dimensions of the collected dental X-ray images varied from 1348 to 2820 pixels in width and 685 to 1348 pixels in height. For consistency of image preprocessing, the images were downsampled to a uniform size of 1200 × 630 pixels, using bilinear interpolation. The final ROI was restricted to the lower part of the mandible, below the teeth-containing alveolar bone, for an image size of 700 × 140 pixels (Figure 1). This included the most ROI areas of previous studies [13,14,15,16,17,18,19,20,21,22,23] that applied various classification techniques by detailed and specifically indexing the image feature characteristics of the limited small region of mandible. By setting the ROI to include most of the mandible instead of the specific area of it, this study evaluated the area that plays the most distinctive role in osteoporosis classification through explainable deep learning techniques.

### 2.3. Convolutional Neural Networks

**Figure 2:**
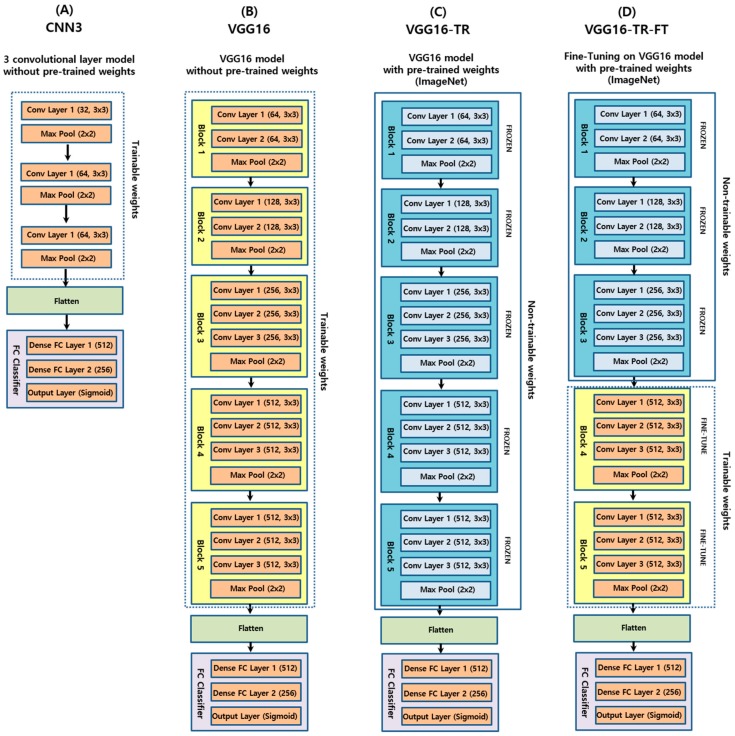
Schematic diagrams of the four convolutional neural networks (CNN) architectures evaluated in this study.

This study employed four study groups of CNN as follows: a basic CNN model with three convolutional layers (CNN3), a visual geometry group deep CNN model with no pre-trained weights (VGG16), a transfer learning model from VGG16 with pre-trained weights (VGG16-TF), and a transfer learning and fine-tuning model from VGG16 with pre-trained weights (VGG16-TF-FT). The preceding architectures, along with the four variant CNN models (CNN3, VGG16, VGG16-TR, and VGG16-TR-FT) used in this study, are depicted in the block diagram in Figure 2.

The reason for choosing VGG16 [31] architecture was that it had been widely adopted and recognized as state-of-the-art in both general and medical image classification tasks [24]. Additionally, it has been trained on large-scale datasets, so that a transfer learning approach could be adopted for large-scale image recognition [38]. For the VGG16 architecture under consideration, the following three different experimental groups were evaluated: the native group (VGG16), transfer learning group (VGG16-TR), and transfer learning with fine-tuning group (VGG16-TR-TF). In the native version, model weights were randomly initialized, and training was conducted using only the DPR data described in this study. In the transfer learning version, model weights were fixed, based on pre-training with a general image dataset, except for the final, fully connected layers, which were randomly initialized. In the transfer learning with fine-tuning version, model weights were initialized based on pre-training on a general image dataset, the same as previous versions, except that some of the last blocks were unfrozen so that their weights were updated in each training step. In this study, the last two transfer learning version models (VGG16-TR and VGG16-TR-FT) employed pre-trained weights using the ImageNet database [38]. ImageNet is an image dataset containing thousands of different objects used to train and evaluate image classification models. 

### 2.4. Model Training

**Figure 3:**
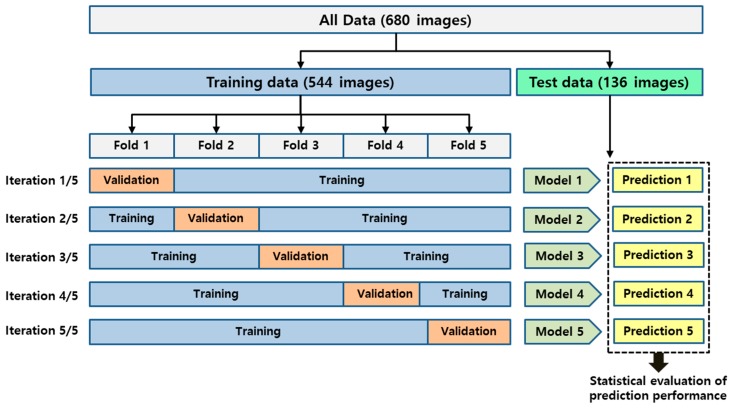
The overview of the performed 5-fold cross validation in this study.

The 544 images selected as the training dataset were randomly divided into five folds. This was done to perform 5-fold cross validation to evaluate the model training, while avoiding overfitting or bias [39]. Within each fold, the dataset was partitioned into independent training and validation sets, using an 80 to 20 percentage split. The selected validation set was a completely independent fold from the other training folds and it was used to evaluate the training status during the training. After one model training step was completed, the other independent fold was used as a validation set and the previous validation set was reused, as part of the training set, to evaluate the model training. An overview of the 5-fold cross validation performed in this study is presented in Figure 3.

This process was repeated for each architecture (CNN3, VGG16, VGG16-TR, and VGG16-TR-FT). All models were trained and evaluated on a 64-bit Windows 10 operating system, with 64 GB memory and an NVIDIA Quadro P4000 GPU. Building, training, validation, and prediction of deep learning models were performed using the Keras [40] library and TensorFlow [41] backend engine.

### 2.5. Performance Evaluation

The evaluation of the screening performance of the CNN models was performed with the independent test dataset in each cross-validation fold. To comprehensively evaluate the screening performance on the test dataset, the accuracy, sensitivity, specificity, receiver operating characteristic (ROC) curve, and precision recall (PR) curve were calculated. The accuracy, sensitivity, and specificity score can be calculated as follows:

$$\mathrm{accuracy}=\frac{\mathrm{TP}+\mathrm{TN}}{\mathrm{TP}+\mathrm{TN}+\mathrm{FN}+\mathrm{FP}}$$

$$\mathrm{sensitivity}=\frac{\mathrm{TP}}{\mathrm{TP}+\mathrm{FN}}$$

$$\mathrm{specificity}=\frac{\mathrm{TN}}{\mathrm{TN}+\mathrm{FP}}\;$$

TP and FP are the number of correctly and incorrectly predicted images, respectively. Similarly, TN and FN represent the number of correctly and incorrectly predicted images, respectively. The area under the ROC curve (AUC) was also calculated.

### 2.6. Visualizing Model Decisions

Deep learning models have often been referred to as non-interpretable black boxes because it is difficult to know the process by which they make predictions. To know the decision-making process of the model, and which features are most important for the model to screen osteoporosis in DPR images, this study employed the gradient-weighted class activation mapping technique (Grad-CAM) [42] and the most significant regions for screening osteoporosis in DPR images were highlighted.

## 3. Results

### 3.1. Baseline Clinical and Demographic Characteristics of the Subjects

The patients were 565 female and 115 male, with an age range from 27 to 90 years (mean age of 63.0 years). There were 380 patients (mean age 58.5) without osteoporosis (T-score ≥ −2.5) and 300 patients (mean age 68.6) with osteoporosis (T-score < −2.5). The clinical characteristics of the DPR dataset used in this study are summarized in Table 1.

### 3.2. Prediction Performance

The CNN models of this study were trained using a cross-entropy loss function on the selected training image dataset. The screening performances of the four CNN models tested in this study are displayed in Table 2. It was observed that the transfer learning and fine tuning VGG16 model with pre-trained weights (VGG16-TR-FT) achieved the top performance, with the highest AUC of 0.858 (95% CI 0.865 to 0.850), sensitivity of 0.900 (95% CI 0.919 to 0.881), specificity of 0.815 (95% CI 0.847 to 0.783), and accuracy of 0.840 (95% CI 0.857 to 0.822). The screening performances of the other models that applied transfer learning techniques, but did not apply fine tuning, one with pre-trained weights (VGG-TR) and the other without pre-trained weights (VGG16), were slightly degraded. The arbitrarily established model with three convolutional layers (CNN3) achieved the lowest performance, with an AUC of 0.667 (95% CI 0.708 to 0.626), sensitivity of 0.684 (95% CI 0.889 to 0.480), specificity of 0.649 (95% CI 0.813 to 0.486), and accuracy of 0.660 (95% CI 0.725 to 0.594).

**Figure 4:**
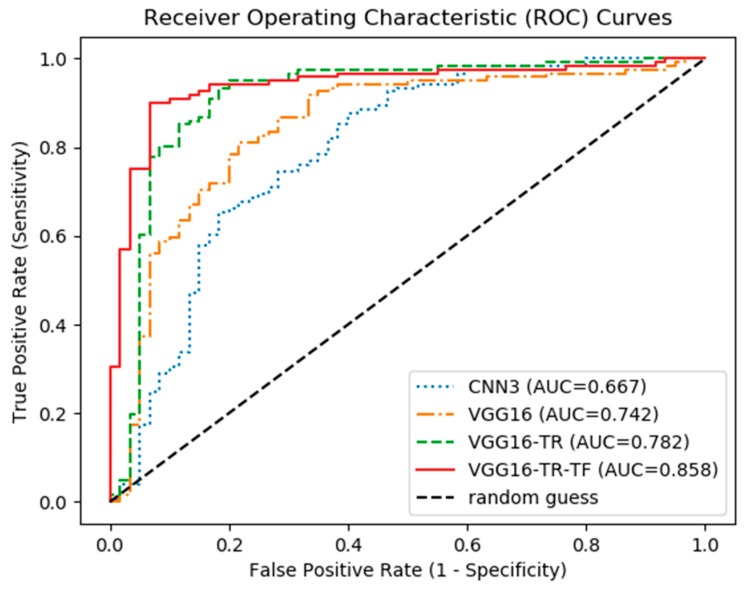
Mean ROC curves of each CNN models for screening osteoporosis on DPR images in this study.

**Figure 5:**
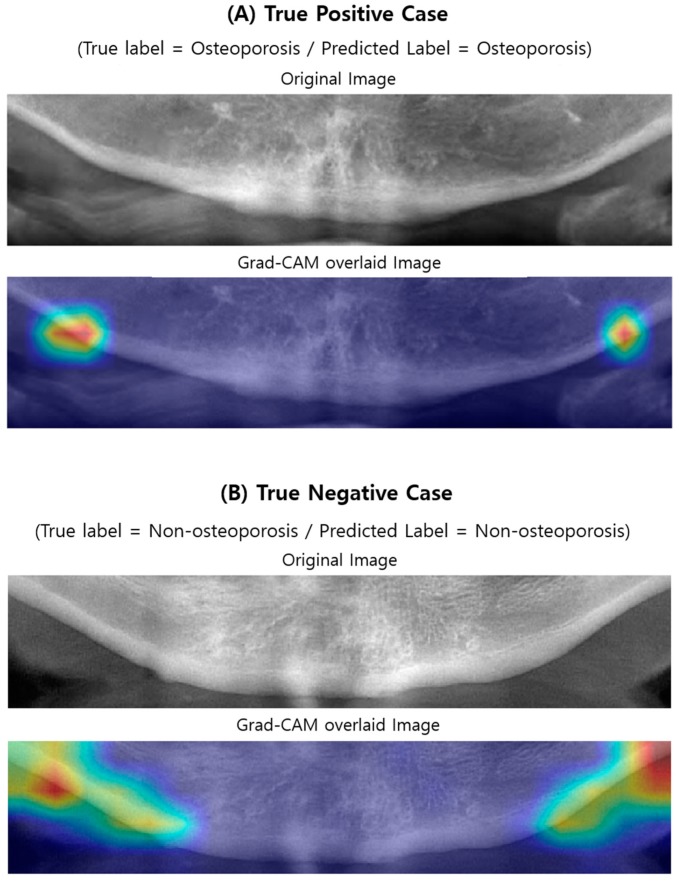
Original and Grad-CAM sample images of correctly predicted by the best-performing deep CNN model (VGG16-TR-TF) for DPR image-based osteoporosis screening are illustrated. Below each original sample images, a Grad-CAM image is superimposed over the original image. The bright red in each Grad-CAM image indicate the region that has the greatest impact on screening osteoporosis patients.

Figure 4 shows the ROC curves of all tested models. The VGG16-TR-FT models achieved the highest AUC of 0.86, while the CNN3 model achieved the lowest AUC of 0.61. Figure 5 shows the PR curves of the tested CNN models. It was also observed that the VGG16-TR-FT models achieved the highest PR of 0.86, while the CNN3 model achieved the lowest PR of 0.61. 

### 3.3. Visualizing Model Decisions

**Figure 6:**
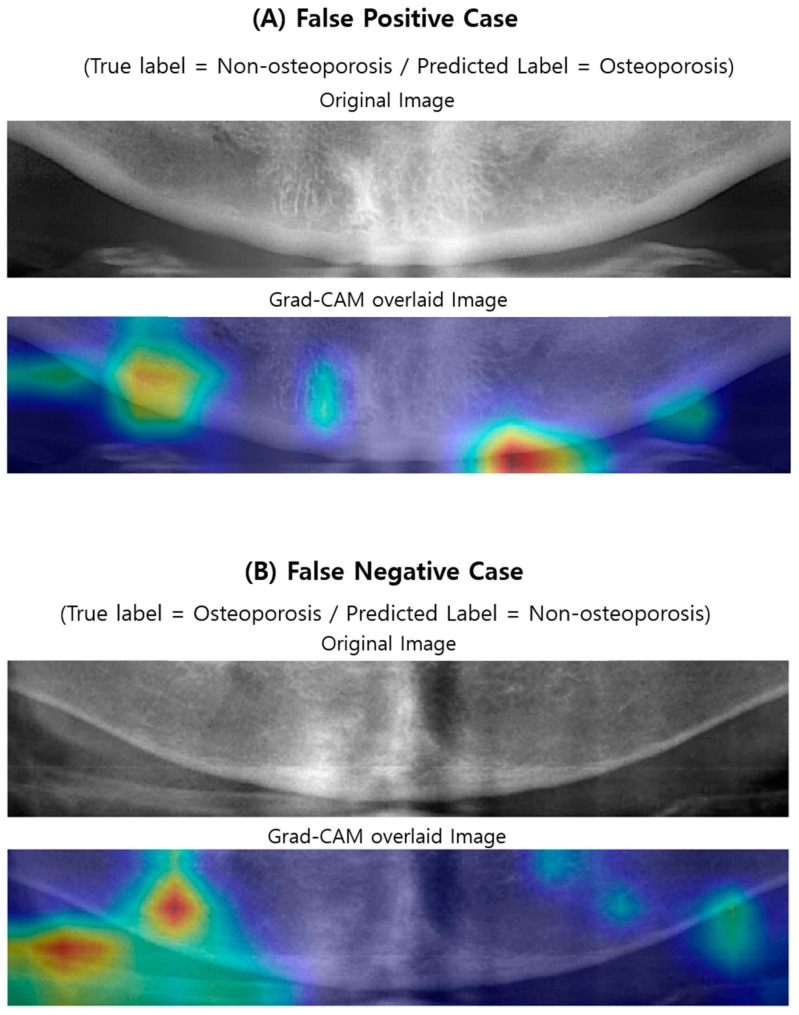
Original and Grad-CAM sample images of incorrectly predicted by the best-performing deep CNN model (VGG16-TR-TF) for DPR image-based osteoporosis screening are illustrated. Below each original sample images, a Grad-CAM image is superimposed over the original image. The bright red in each Grad-CAM image indicate the region that has the greatest impact on screening osteoporosis patients.

Figure 5 and Figure 6 illustrate the case examples of predictions using the best predictive VGG16-TR-FT model as compared with ground truth. Each case example employed a Grad-CAM technique to perform a visual interpretation to determine which areas affected the deep CNN’s class classification. In the case of screening correctly for osteoporosis (Figure 5A), the region showing the weak lower border of the mandibular cortical bone and the less dense, spongy bone texture at its periphery was extracted as the main image feature of the classification. In correctly screened cases of no osteoporosis (Figure 5B), the region showing the strong lower boundary of the mandible cortical bone and the dense texture around its periphery was extracted as the main image feature of the classification. However, in the case of incorrectly screened cases, i.e., the non-osteoporosis case predicted as osteoporosis (Figure 6A) or the osteoporosis case predicted as non-osteoporosis (Figure 6B), the central region of the mandible or the ghost images of the hyoid bone was extracted as the main image feature.

## 4. Discussion

Although DPRs are commonly performed for the evaluation of dentition and adjacent structures of the jaw, some clinical assistant diagnosis (CAD) systems based on DPRs have been suggested for screening systemic diseases, such as osteoporosis and carotid artery calcification [13,14,15,16,17,18,19,20,21,22,23,43]. However, the approaches of most previous studies are only valid when image features are accurately extracted, using sophisticated and manual image preprocessing algorithms or techniques. If a DPR image is imported from an unfamiliar environment or unexpected noise is added to the image, the prediction can easily be distorted. The neural network algorithm can resolve this problem. All the knowledge necessary for diagnosis is established only with the given training image data, without complicated or sophisticated image preprocessing. In recent years, a cutting-edge neural network technology, called deep learning, has been applied to medical imaging analysis and has shown a level of performance that is equal to or better than a clinician. As mentioned above, most previous CAD system studies, which used manual or sophisticated image preprocessing and machine learning algorithms for the screening of osteoporosis based on DPRs, presented variable diagnostic performances, in terms of sensitivity and specificity [13,14,15,16,17,18,19,20,21,22,23]. Recently, a deep learning-based osteoporosis prescreening study, which resulted in a very high AUC score (0.9763 to 0.9991) and accuracy (92.5% to 98.5%), was published [44]. However, in that study, osteoporosis labeling was subjectively performed by dental specialists, rather than BMD score (T-score) which is the gold standard for diagnosing osteoporosis. In addition, the study did not visually interpret the decision of the trained CNN model, and using five arbitrarily established convolutional layers, there is a limitation to the reproducibility of the deep CNN model.

The first major findings of the present study showed that applying appropriate transfer learning and fine-tuning techniques on pre-trained deep CNN architectures had an equivalent DPR-based osteoporosis screening level of previous studies, even with small image datasets, without complex image preprocessing and image ROI settings. According to Table 2 and Figure 4, the CNN3 group, having only arbitrary established three convolutional layers, showed the lowest true-positive screening performance and accuracy among the experimental groups. On the basis of these results, it can be estimated that a CNN model with a small number of convolutional layers can have limitation in learning the true data distribution from a small number of datasets. 

Comparing models that used pre-trained weights (VGG16-TR and VGG16-TR-FT) to those that did not (VGG16), also revealed that deep CNNs initialized with large-scale pre-trained weights outperformed those directly learnt from small-scale data, with AUC improvements between 7% to 11%. Thus, in the case of having a small-scale image dataset, this study also suggests that the use of transfer learning on deep CNN models with pre-trained weights can be an efficient solution for the classification of medical images, instead of learning a deep neural network from scratch.

**Figure 7:**
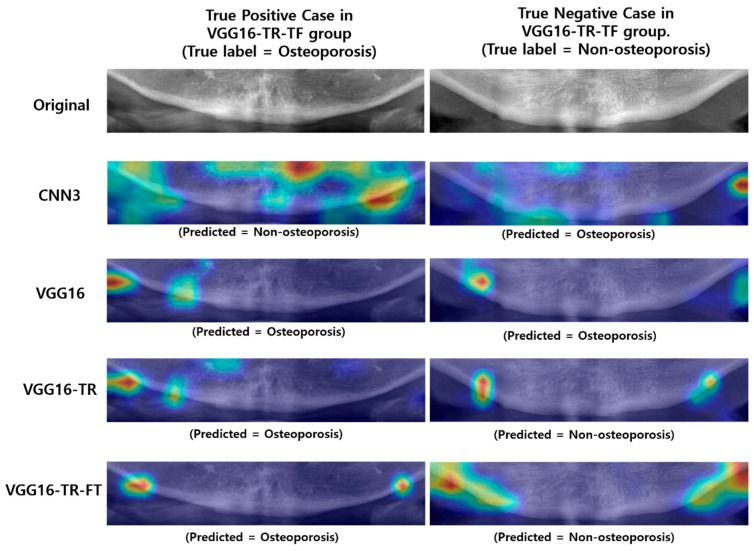
Comparison of grad-CAM images from other groups against some original images showing true positive and true negative in the best performing VGG16-TR-TF group.

**Figure 8:**
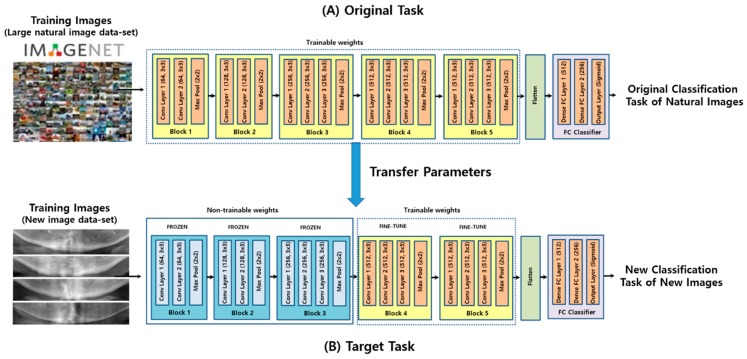
The conceptual diagram of the fine-tuning technique in the transfer learning of a deep CNN.

Moreover, as shown in Table 2 and Figure 7, the results of this study also indicated an improvement in screening performance when using fine-tuning on some convolutional blocks in deep CNN layers. In general, the deep CNN model learned from pre-trained deep neural networks on a large natural image dataset could be used to classify common images but cannot be well utilized for specific classifying tasks of medical images (Figure 8A). However, according to a previous study that described the effects and mechanisms of fine tuning on deep CNNs [45], when certain convolutional blocks of a deep CNN model were fine-tuned, the deep CNN model could be further specialized for specific classifying tasks (Figure 8B). More specifically, earlier layers of a deep CNN contain generic features that should be useful to many classification tasks, but later layers progressively contain more specialized features to the details of the classes contained in the original dataset (i.e., the large natural image dataset on which the deep CNN was originally trained). Using this property, when the parameters of the early layers are preserved and the parameters in later layers are updated during training new datasets, the deep CNN model can be effectively used in new classification tasks. In conclusion, fine-tuning uses the parameters learned from a previous training of the network on a large dataset and, then, adjusts the parameters in later layers from the new dataset, improving the performance and accuracy in the new classification task. As with the previous study, the fine-tuning technique, which freezes the weight parameters of some initial convolutional blocks in the deep CNN model called VGG16, and, then, updates the weight parameters of the later convolutional blocks (Figure 8B), show higher performance than other experimental groups. The conceptual diagram of the fine-tuning technique mentioned above can be seen in Figure 8.

The second major result of this study was to identify areas where image feature differences occurred when screening osteoporosis in DPR images using the Grad-CAM technique. To understand and visualize the decision of deep CNN models, some samples of the correctly and incorrectly screened examples were reviewed (Figure 5 and Figure 6). For additional insight to model decisions, a Grad-CAM technique was performed in this study. This technique identified the areas of input images that had the greatest impact on model classification. According to this additional review, the model does seem to identify the feature characteristics of osteoporosis in DPR images (e.g., cortical bone thinning). According to the Grad-CAM evaluation of this study, DPR-based screening performances of osteoporosis were high when the image features were specified in the middle region of the left and right side of the mandibular lower border. This region is also consistent with the regions used to discriminate osteoporosis using DPR images, in most previous studies [13,14,15,16,17,18,19,20,21,22,23], although the measurement algorithm was different. This indicates that most osteoporosis patients have image feature characteristics, on DPR images, at the lower border of the cortical bone in the mandible. However, image quality issues, such as blurring, low contrast, and ghost images of adjacent objects can cause incorrect predictions. When the image features were specified in the center region of the mandible, or when the ghost images of the hyoid bone were in the ROI region, the accuracy was reduced. Therefore, to improve the deep CNN-based screening performance of osteoporosis in DPR images, it is suggested that the ROI setting be limited to the area around the middle of the left and right side of the lower border of the mandible.

## 5. Conclusions

This study presents the usefulness of transfer learning and fine tuning with a deep CNN for the screening of osteoporosis in DPR images, in cases with a limited training dataset. We have applied various transfer learning techniques on pre-trained networks VGG16 for the discrimination of osteoporosis using a DPR image dataset, labeled based on T-score. The experimental results showed that transfer learning with pre-trained weights and fine-tuning techniques achieved the highest overall accuracy of 84%. The presented results suggest that the combination of the appropriate deep CNN architectures and transfer learning techniques has effectively resolved the issue of a small training set of images and that DPR images have the potential for osteoporosis prescreening. In addition, using the Grad-CAM technique, this study performed a deep learning-based visual explanation for the area where the image feature difference occurred. Therefore, this study confirmed the previous osteoporosis screening studies using DPR images that set the ROI at the middle of the left and right side of the lower border of the mandible. Given the increasing burden of osteoporosis on the global healthcare system, as our population ages, and the proliferation of dental panoramic image devices, the results presented in this study suggest that deep learning-based image analysis of DPRs could serve an important role in cost-effective prescreening for patients unaware of osteoporosis. To further improve screening performance, future research is needed, using different deep CNN architectures and deep learning techniques, more validated and qualified labeled image dataset, the appropriate number of datasets, and automated configuration techniques for more limited range of ROI.

## Figures and Tables

**Table 1:** Clinical and demographic characteristics of the dental panorama radiographs (DPRs) dataset in this study.

Parameter	Without Osteoporosis (T-Score ≥ −2.5)	With Osteoporosis (T-Score < −2.5)	Total
Number of patients	380	300	680
Number of female/male	332/48	233/67	565/115
Mean age (±SD)	58.5 (±11.8)	68.4 (±8.4)	63.0 (±11.6)

**Table 2:** Osteoporosis screening accuracy of convolutional neural network models in this research.

Model	AUC (95% CI)	Sensitivity (95% CI)	Specificity (95% CI)	Accuracy (95% CI)
CNN3	0.667 (±0.041)	0.684 (±0.204)	0.649 (±0.164)	0.660 (±0.066)
VGG16	0.742 (±0.018)	0.674 (±0.048)	0.811 (±0.034)	0.771 (±0.018)
VGG16-TR	0.782 (±0.006)	0.737 (±0.046)	0.828 (±0.052)	0.802 (±0.024)
VGG16-TR-TF	0.858 (±0.008)	0.900 (±0.019)	0.815 (±0.032)	0.840 (±0.018)
